# Cardiovascular physical examination as a screening tool for congenital heart disease in newborns at a teaching hospital in Ghana

**DOI:** 10.4314/gmj.v57i2.10

**Published:** 2023-06

**Authors:** Frank Owusu-Sekyere, Bamenla Goka, Della Adzosii, William Obeng, Alfred Yawson, Nana Akyaa-Yao, Sybil Harrison, Justice Moses K. Aheto

**Affiliations:** 1 Department of Child Health, Korle Bu Teaching Hospital, Accra, Ghana; 2 Department of Biostatistics, School of Public Health, University of Ghana, Legon Ghana; 3 Department of Child Health, University of Ghana Medical School, Accra, Ghana; 4 Korle Bu Teaching Hospital, Accra, Ghana; 5 National Cardiothoracic Centre, Korle Bu Teaching Hospital, Accra, Ghana

**Keywords:** Congenital heart disease, cardiovascular physical examination, screening tool, newborns, Korle Bu Teaching Hospital

## Abstract

**Objectives:**

To determine the usefulness of cardiovascular physical examination (CPE) as a screening tool in a low-resource setting for detecting congenital heart disease (CHD) in newborns delivered at the Maternity Unit of Korle Bu Teaching Hospital (KBTH), Accra, Ghana.

**Design:**

A hospital-based cross-sectional study with a comparison group component.

**Setting:**

Maternity Unit of the KBTH, Accra, Ghana.

**Participants:**

Over eight months, newborns aged 1-14 days delivered at ≥ 34 weeks' gestation at the Maternity Unit, KBTH, were recruited into the study.

**Intervention:**

Each newborn was examined using a set of CPE parameters for the presence of congenital heart disease. Those with suggestive features of CHD had a confirmatory echocardiogram test.

**Main Outcome Measure:**

Abnormal CPE features and their corresponding echocardiogram findings.

**Results:**

A total of 1607 were screened, with 52 newborns showing signs of CHD on CPE, of which 20 newborns were proven on echocardiogram to have congenital heart disease. Abnormal CPE parameter that was associated with CHD was murmur (P=0.001), dysmorphism (p=0.01), newborns with chest recessions (p=0.01) and lethargy (p=0.02). CPE's sensitivity, specificity, and positive and negative predictive values were 95%, 60.7%, 36.5% and 98,1%, respectively. The most common acyanotic CHD found was isolated atrial septal defect (ASD), followed by patent ductus arteriosus (PDA). The only cyanotic CHD found was a case of tricuspid atresia.

**Conclusion:**

Cardiovascular physical examination at birth is an effective and inexpensive screening tool for detecting CHD in newborns, which can easily be utilised in low-resource settings.

**Funding:**

None declared.

## Introduction

Congenital heart disease (CHD) is the commonest group of congenital malformations and contributes significantly to childhood morbidity and mortality worldwide. It encompasses a heterogeneous group of congenital cardiac defects, including critical congenital heart diseases (CCHD), in which surgical or interventional therapy within the first year of life is mandatory to achieve survival.^1^,^2^ The birth prevalence of CHD is similar worldwide, with little variations between regions due to genetic, environmental, and epigenetic differences.^3^

The estimate of 8 per 1000 live births^1^ is generally accepted as the most reliable even though studies from postnatal nurseries have found higher values of 12 – 14/1000 live births.^4^

Congenital Heart disease was thought to be rare in Africa. In a systematic review of PubMed literature by van der Linde et al., Africa had the lowest prevalence rate for CHD of 1.9/1000. 5 However, in 1967, a landmark study done in Nigeria, using clinical features of heart defects, radiological evidence and electrocardiography as well as necropsy findings, found a prevalence rate of 3.5 per 1000 live birth.^6^ A comparatively recent study done at the University of Benin Teaching Hospital in Nigeria using clinical, radiographical, electrocardiogram and echocardiogram features, found a prevalence of 4.6 per 1000 live births.^7^

In Ghana, CHD's exact prevalence and relative distribution are poorly documented. However, a five-year review of echocardiograms done at the National Cardiothoracic Centre in Ghana from January 2006 to December 2010 found that out of 4,823 echocardiograms done, 60.3% were due to newly diagnosed CHD.^8^

Reported prevalence studies for CHD have shown wide variability worldwide, including studies done in Africa. The variability depends on when the study was done, the study design and whether the study stratified the CHD findings into trivial, moderate or severe forms.^1^,^2^,^4^,^6^.

Congenital heart disease may be asymptomatic at birth, particularly in cases of CCHD. Thus, the importance of early detection and treatment cannot be overemphasised. Also, congenital heart defects may not be easily recognised at birth or soon after until complications occur. Screening helps to detect such lesions early before clinical deterioration occurs.^9^

Timely diagnosis of CHD, particularly the critical lesions, remains a challenge even in well-resourced countries.^10^ Babies with CCHD often present as an emergency when the ductus arteriosus closes with loss of systemic vascular perfusion and severe cyanosis.^10^ Screening and early diagnosis, together with early interventions, help to reduce mortality and manage better the associated morbidity.

Historically, screening for CHD relied on physical examination with follow-up of those at significant risk. Also, chest X-rays and electrocardiograms (ECG) have been used as screening tests for congenital heart diseases.^11^ Over time, in addition to the above, other methods have been employed as screening tools to detect CHD. These include a prenatal ultrasound scan,^12^,^13^ pulse oximetry^14^,^15^ and a combination of these methods.^16^,^17^ Those suspected to have CHD on screening would require a confirmatory test. Cardiac catheterization^18^ and echocardiography^19^ have been used as confirmatory tests. Because of its non-invasiveness, echocardiography is preferred and has been the gold standard for confirmation of congenital heart diseases.^19^ CHD is believed to be common in Ghana.

Edwin *et al.* report that of the 100-120 cardiac surgeries done annually at KBTH in Ghana, 25% had CHD.^20^ Also, unpublished data shows an increasing number of late diagnoses of children with CHD at the Department of Child Health, KBTH. This underscores the need for early detection and treatment of CHD in Ghana. Cardiovascular physical examination and pulse oximetry for screening heart diseases have been extensively used to diagnose CHD early in many countries.^15^,^21^ However, as is the case in many developing countries, there is a paucity of data regarding screening for congenital heart diseases using these methods in Ghana.

In Ghana, expertise for prenatal ultrasound cardiac diagnosis is in its infantile stages and cardiovascular physical examination is not routinely done for all newborns. This study, therefore, sought to validate cardiovascular physical examination as a screening tool for CHD in a low-resource setting.

## Methods

### Study design and setting

This is a cross-sectional hospital-based study with a comparison group conducted at the Maternity Unit of KBTH, the largest tertiary referral centre in Ghana and the largest in the number of deliveries per year in Ghana.

### Study population

All newborns delivered at the Maternity Unit of KBTH between August 2015 and March 2016 were recruited into the study.

**Inclusion criteria:** All newborns at ≥ 34 weeks' gestation and aged 24 hours to 14 days of life.

**Exclusion criteria:** Preterms below 34 weeks gestation were excluded.

### Data Collection

The study was conducted between August 2015 and March 2016 to consecutively recruit newborns delivered at KBTH from the Neonatal Intensive Care Unit (NICU), postnatal wards and postnatal clinics between 24 hours and 14 days. All these babies had already been examined by the Obstetrics team and were declared to have no CHD or referred to NICU for other reasons. Written informed consent was sought from and signed by the mothers, and all newborns over 34 weeks gestation period were included unless the mother opted out. All data were collected per the approved ethical clearance numbered MSEt/M.1-P4.5/2014-2015 from The Ethical and Protocol Review Committee of the University of Ghana Medical School.

Baseline information of gestational age, age in days, sex, birth weight, mode of delivery and APGAR scores were recorded. The newborns were examined for evidence of congenital heart disease based on cardiovascular physical examination (CPE) using the following set of parameters: tachypnoea (respirator rate > 60 cycles/min) - unexplained or with feeding, chest retractions- subcostal, intercostal, tracheal tug, central cyanosis (bluish discolouration of mouth and tongue with white light), active precordium, weak or absent peripheral pulses, cardiac dextroposition, unexplained tachycardia (Heart rate of > 160 b/min), irregular heartbeats, heart murmurs, capillary refill time (CRT) > 2 seconds, tender hepatomegaly (> 3cm below the costal margin in the presence of tachypnoea and tachycardia) and dysmorphia. Every participant had the heart sounds and the presence or otherwise of murmurs documented. Those with murmurs had characteristics such as type, the location where the murmur was maximally heard, its radiation, and the grade (graded out of six) documented.

In this study, preterm babies, especially those below 34 weeks, frequently have PDA. It would have been difficult to tell which is abnormal or normal; hence, babies below 34 weeks gestation were excluded. Every baby who satisfied a positive criterion on CPE had a transthoracic echocardiogram done by a paediatric cardiologist using a Phillips Matrix CX50 machine for confirmatory evidence of CHD. The subcostal, right anterior oblique, parasternal long and short axis, apical four-chamber, aortic arch and ductal views were used to image the heart. The colour-doppler was used to ascertain flow direction and M-mode for assessing the heart's function.

A consecutive recruitment procedure was employed to recruit the participants over the period between August 2015 to March 2016. During this period, 1607 newborns were screened for evidence of CHD via CPE; hence a total of 1607 newborns were screened, out of which the required sample of 52 showed evidence of CHD on screening by CPE (cases). All the babies were examined by one doctor specialising in paediatric cardiology. The difference between our screening test in this study and the routine examination conducted at the hospital is that our study employed a set of clinical parameters specifically aimed at detecting CHD.

Newborns who showed evidence of CHD on screening by CPE were categorised as Positive Screens (i.e., cases). For validating CPE as a screening tool, an equal number of newborns (52) with no evidence of CHD on CPE and categorised as Negative Screens were randomly selected. An echocardiogram was done, serving as a comparison group. The validity of the screening method (i.e., CPE) was determined using sensitivity and specificity, including positive and negative predicted values.

The screening on echocardiogram was considered the ‘Gold standard’ while the screening on CPE was considered the screening test.

### Data Analyses

Data entry, re-coding, validation, and analysis were performed using Statistical Package for Social Sciences (SPSS) version 22. Demographic characteristics of babies and data on cardiovascular physical examination (including tachypnoea with feeding, central cyanosis, chest retractions, active precordium, weak or absent peripheral pulses, cardiac dextroposition, unexplained tachycardia) were summarised using descriptive statistics. Categorical variables were summarised using frequencies and their associated percentages, while means and their associated standard deviations were used to summarise continuous variables.

Significant associations between the categorical predictor variables and CHD in newborns were analysed with the Chi-squared test of independence, except where any expected frequencies for a particular cell was less than 5, Fisher's Exact test was applied. The test of differences in means for continuous predictor variables (e.g., heart rate and pulse rate) against CHD status were determined by independent t-test for group means. The sensitivity, specificity, and negative predictive values were estimated to determine how well the CPE correctly identifies new-borns with positive or negative CHD. To estimate these values, we let *A* represents the number of true positives, *B* is the number of false positives, *C* is the number of false negatives, and *D* is the number of true negatives. Thus, we estimate these quantities as sensitivity = (*A*/(*A*+*C*))*100, specificity = (*D*/(*B*+*D*))*100, positive predictive value = (*A*/*A*+*B*)*100, and negative predictive value = (*D*/(*C*+*D*))*100. Significant associations were determined at a p-value <0.05.

## Results

**Figure 1:**
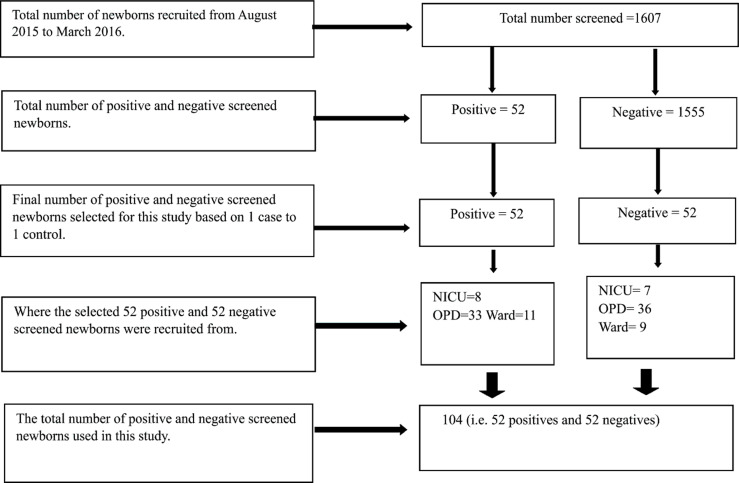
The recruitment procedure and final samples used in this study. NICU=Neonatal Intensive Care Unit

Figure 1 shows the screening and recruitment processes undertaken in this study. A total of 1607 newborns were screened consecutively until the required sample of 52 cases was identified. Because we proposed to do 1 case to 1 control, we applied a simple random sampling approach to select 52 controls from the 1555 negatives, making a final sample size of 104 for the study.

The mean gestational age is 39 weeks (±1.8 weeks), and nearly 48% of babies were delivered through caesarean section (CS). There was slight male preponderance. Table 1 summarises the baseline characteristics of study participants.

**Table 1:** Baseline characteristics of all newborns screened for CHD

Variables	N=1607
**Mean gestational age (weeks)**	39.(±1.8)
**Mode of delivery**	
**CS**	768 (47.8%)
**SVD**	834 (51.9%)
**Vacuum**	5 (0.3%)
**Sex**	
**Male**	810 (50.4%)
**Female**	797 (49.6%)
**Mean Birth weight (kg)**	2.96 (±0.8)
**Apgar scores**	
**1 minute**	7 (±1)
**5 minutes**	8 (±1)
**Mean age at recruitment (days)**	11 (±3.7)
**Location of recruitment**	
**Postnatal Clinic**	882 (54.9%)
**Ward**	462 (28.7%)
**NICU**	263 (16.4%)

SVD=Spontaneous Vaginal Delivery, CS=Caesarean Section, OPD=Outpatient department, NICU =Neonatal Intensive Care Unit

All 1607 newborns examined were well perfused with a capillary refill time of <2s. All had strong peripheral pulses, including the femoral, quiet precordia, with their apex located in the fourth intercostal space in the left hemithorax. Only one newborn was breathless with feeding. Two babies had hepatomegaly in the presence of tachypnoea and tachycardia. Selected examination findings are presented in Table 2.

**Table 2:** Selected physical examination findings of all newborns (N=1607)

Features	N(%)
**Alert**	1603 (99.8)
**Lethargic**	4 (0.2)
**Dysmorphic features**	
**No**	1604 (99.8)
**Yes**	3 (0.2)
**Cyanosis**	
**No**	1605 (99.9)
**Yes**	2 (0.1)
**Murmurs**	
**No**	1558 (97.0)
**Yes**	49 (3.0)
**Respiratory distress**	
**No**	1602 (99.7)
**Yes**	5 (0.3)

All four lethargic babies shown in Table 2 were already on admission at the NICU, and two of these four had murmurs. Of the three babies with dysmorphic features, one had facies consistent with Down Syndrome, one had microcephaly, microphthalmia, and bilateral cataract consistent with Congenital Rubella Syndrome, and the third had micrognathia.

Two babies with cyanosis were already on admission to NICU and were on intranasal oxygen. Five babies with signs of respiratory distress had flaring of the *alae nasi*, subcostal and intercostal recessions. Only one, however, had tracheal tug as well, and all five were on admission to NICU. Some babies had a combination of these features; for example, the baby with micrognathia was clinically cyanosed, lethargic and had flaring of the *alae nasi*, subcostal and intercostal recessions, and a tracheal tug.

A heart murmur was the most common abnormal sign on CPE among the newborns examined. Of the 52 newborns with abnormal CPE, heart murmur, 49(94.2%) was the commonest abnormal sign. By way of murmur types, 34 (69.4%) of murmurs heard were pan systolic, 13(26.5%) were ejection systolic, and 2(4.1%) were machinery murmurs. Some babies had other abnormal CPE features in addition to murmurs. For example, one baby was cyanosed with signs of respiratory distress and had a murmur. The commonest type of murmur heard was pan systolic; the commonest location where the murmur was maximally heard was the lower left sternal edge. One baby with an ejection systolic murmur at the pulmonary area had the murmur radiating to the back. None of the babies had a murmur beyond grade 3 out of 6. Nineteen (19) out of the 49 babies with murmurs (39%) had CHD. A total of 104 newborns comprising 52 Positive Screens and 52 Negative Screens, had echocardiograms done to examine for CHD. Twenty (20) newborns had CHD giving a prevalence of CHD of 12.4 per 1000 live birth in this study.

The mean gestational age of the Positive Screens was significantly higher (39.3 ± 1.8) weeks compared to 38.5 ± 2.2 weeks of the Negative screens (p=0.04). Both Positive (n=52) and Negative Screens (n=52) had comparable mean birth weight, mode of delivery, sex distribution, age at enrolment into the study and Apgar scores. A comparison of other parameters did not show any significant differences, as shown in Table 3.

**Table 3:** Comparison of some baseline clinical characteristics of Positive Screens and Negative screens

Characteristics	Positive Screen (N=52)	Negative Screen (N=52)	p-value
**Mean Gestational age (Weeks)**	39.3 (±1.8)	38.5 (±2.2)	0.04^a^
**Mode of delivery**			
**C/S**	24	24	
**SVD**	28	28	1.00^b^
**Sex**			
**Male**	31	26	
**Female**	21	26	0.33^b^
**Mean age at recruitment (days)**	11.2 (±4.7)	10.3 (±4.8)	0.35^a^
**Mean birth weight (kg)**	3.1 (±0.9)	3.3 (±0.7)	0.97^a^
**Apgar scores**			
**1 minute**	7 (±1.3)	6.7 (±1.7)	0.97^a^
**5 minutes**	8 (±1.0)	8.2 (±1.2)	0.59^a^
**Mean respiratory rate**	49.0 (±11.1)	50.6 (±6.5)	0.39^a^
**Mean apex rate**	134.4 (±21.0)	137.3 (±10.5)	0.38^a^

SVD=Spontaneous Vaginal Delivery; CS=Caesarean Section; a=p-value for independent t-test; b=p-value for Pearson chi-square test

Nineteen (36.5%) out of the 52 Positive Screens were diagnosed with various forms of CHD and one newborn from the Negative Screens had CHD (ASD), making a total of 20 confirmed CHD newborns. Of the 20 confirmed CHD newborns, 19 (95%) were of the acyanotic type, and 1 (5%) was cyanotic.

Presented in Table 4 are the echocardiogram findings among the Positive and Negative Screens. Six (6) babies with ASD were from the Positive Screens, 5 (83%) were of the ostium secundum type, and 1 (17%) was of the *primum* type. Four of the five babies with the ostium secundum type were of moderate size measuring 7mm, and the fifth was of small size, measuring 4mm. The ostium *primum* type was small-sized, measuring 4mm.

**Table 4:** Echocardiogram Findings among Positive and Negative Screens

Screens	Echocardiogram	
	Positive Screens	Negative Screens
	N(%)	N(%)
**Normal**	33 (63.5)	51 (98.1)
**ASD**	6 (11.5)	1 (1.9)
**PDA**	5 (9.6)	0 (0.0)
**VSD**	3 (5.8)	0 (0.0)
**VSD +PDA**	1 (1.9)	0 (0.0)
**Branch pulmonary artery hypoplasia**	2 (3.8)	0 (0.0)
**Pulmonary stenosis**	1 (1.9)	0 (0.0)
**Tricuspid atresia**	1 (1.9)	0 (0.0)
**Total**	52 (100.0)	52 (100.0)

ASD-Atrial septal defect; PDA-Patent ductus arteriosus; VSD-Ventricular septal defect.

Three (60%) of the five babies with patent ductus arteriosus (PDA) were of moderate to large size with dilatation of the left side of the heart, 2 (40%) were small-sized and restrictive. The gestational age range for all babies with PDA was 38 – 41 weeks.

A total of 3 babies had isolated ventricular septal defects (VSD). Two were of the muscular type, and one was peri-membranous. One of the two muscular types was small, and the other was moderate, with volume loading of the left side of the heart. The third baby with isolated VSD had a large peri-membranous type with volume loading of the left heart. One baby had a large inlet-type VSD and a PDA with volume loading of the left side of the heart.

Three babies had anomalies of the pulmonary arteries, one had pulmonary stenosis, and two had branch pulmonary artery hypoplasia. One baby with branch pulmonary artery hypoplasia was the only one with a murmur radiating to the back. The only CCHD was a case of tricuspid atresia. Out of the 52 Negative Screens, one baby had moderate-sized ostium secundum ASD of 6mm. Echocardiogram revealed no cardiac pathology in the other 51 (98.1%) babies.

A total of 20 babies, 19 from the Positive Screens and one from the Negative Screens, had CHD. Thus, the sensitivity (95%CI), specificity (95%CI), positive predictive (95%CI), and negative predictive values(95%CI) for CPE as a screening tool for CHD in this study were 95.0% (75%,100%), 60.7% (49%,71%), 36.5% (24%,51%) and 98.1% (90%,100%) respectively. In this study, cardiovascular physical examination findings associated with CHD are lethargy, dysmorphism, chest recessions and murmurs, as shown in Table 5.

**Table 5:** Comparison of some examination findings of newborns with CHD and those without CHD

Variables	CHD (N=20)	Normal (N=84)	P-value
**Lethargy**			
**No**	17	83	
**Yes**	3	1	0.02^a^
**Dysmorphic signs**			
**No**	17	84	
**Yes**	3	0	0.01^a^
**Cyanosis**			
**No**	19	83	
**Yes**	1	1	0.35^a^
**Chest recession**			
**No**	16	83	
**Yes**	4	1	0.01^a^
**Breathless when feeding**			
**No**	19	84	
**Yes**	1	0	0.19^a^
**Murmur**			
**No**	1	54	
**Yes**	19	30	0.001^a^
**Respiratory rate**	53.4 (±12.4)	49.0 (±7.9)	0.05^b^
**Apex rate**	135.1 (±14.3)	136.1 (±17.1)	0.81^b^

a=p-value for Fisher's exact test; b=p-value for independent t-test; CHD= congenital heart disease

## Discussion

The study evaluated CPE as an effective screening tool for CHD examination among newborns. Although CHD is present at birth, obvious signs and symptoms may not be present until clinical deterioration sets in. A neonatal heart murmur is the most common reason for paediatric cardiologist consultation in neonatal intensive care units and nurseries.^22^ This study supports the finding that a murmur is the most common abnormal clinical sign associated with CHD.^23^ Irrespective of other symptoms, a heart murmur was the commonest abnormal physical finding significantly associated with CHD in this study. Studies have shown a 51.4%-54% chance of an underlying cardiac malformation when a murmur is heard in a neonate.^24^, ^25^ This percentage in our study was 36% which was lower. Comparatively fewer patients screened in this study could have accounted for the observed difference.

Newborns with CHD can present with respiratory distress, particularly those with non-restrictive pulmonary blood flow, such as large PDA and transposition of the great arteries. The finding of respiratory distress as evidenced by chest recessions observed as a significant finding from the present study is consistent with the literature.^26^ However, cyanosis, which has been described as the most dramatic sign in newborns that warrants ruling out CHD, was found to be an insignificant association with CHD in this study, a finding not consistent with results of other studies where cyanosis was a significant predictor of CHD.^27^,^28^ The methodology Vaidyanathan et al^28^ used of initial screening at birth and re-screening of those found to be normal on initial screening at six weeks may have picked up some babies with duct-dependent lesions whose visible cyanosis appeared later. Furthermore, the higher number of 5487 screened by Vaidyanathan *et al* may account for their observed significant association of cyanosis and CHD. The comparatively lower sample size (about a third) coupled with the one-off screening in this study may account for the low number of babies with cyanosis observed. Also, most (66%) cases were recruited from the OPD on day 14 because they were discharged home before 24 hours of age. There is a possibility that some babies with cyanosis may have been missed because they died from CCHD before their 14^th^-day OPD review to be recruited. A study design where babies are tracked after discharge to ascertain their outcomes could be warranted to address this issue.

The link between chromosomal abnormalities manifesting phenotypically as syndromes or dysmorphism and CHD is well known. Meberg *et al* observed an association between babies with chromosomal anomalies and CHD.^29^ The present study found a significant association between dysmorphism and CHD. However, the 100% (all three dysmorphic babies had CHD) association of dysmorphic babies and CHD observed in the present study is higher than the 31% found by Meberg *et al.*^29^ The fewer numbers of dysmorphic babies seen in this study could have skewed the percentage. Due to the small number of babies with dysmorphic features in this study, it may be misleading to draw definitive conclusions, as many syndromes with dysmorphic features do not have cardiovascular involvement. However, the findings from this study, support the standard practice of echocardiogram for babies with dysmorphism.

Lethargy as a physical sign in newborns has been associated with CHD^30^ even though it could be from various causes - birth asphyxia, intracranial haemorrhage, sepsis, inborn errors of metabolism and CHD, among others. This study showed a significant association between lethargy and CHD. The methodology employed in this study could not exclude other common causes of lethargy, such as metabolic conditions, sepsis, or babies with intracranial haemorrhage, among others; hence this finding may need to be interpreted cautiously.

A cardiovascular physical examination as a screening tool has been extensively studied and routinely used in most hospitals in well-resourced countries.^11^ Its advantage lies in that house officers or nurse practitioners, not necessarily senior doctors or cardiologists, can perform it.^11^ The sensitivity and specificity from this study for CPE as a screening test for CHD were 95% and 60.7%, respectively. This high sensitivity is comparable to 94% by Gengsakul *et al*, who studied the accuracy of physical examination utilising echocardiography as the gold standard to detect CHD in 500 babies in Thailand.^31^ Similarly, Albuquerque *et al.*, who used cyanosis, precordial palpation, peripheral pulses, and heart murmur as CHD screening tools, recorded a high sensitivity of 88.8%.^32^ The high sensitivity realised from this study, however, differed from that of Bakr *et al.*, who recorded 46% sensitivity with clinical examination alone as a screening test for CHD.^17^ The method in the current study of not attempting to judge whether some murmurs were innocent or pathological helped to reduce the number of occasions where a structural defect was missed.

The moderate specificity of CPE from this study of 60.7% was lower than 98% from Gengsakul *et al*^31^ and 99.7% from Albuquerque *et al.*^32^. The positive predictive value for CPE of 36.5% was comparable to that of Albuquerque *et al's*^32^ but lower than the 51.5% from Gengsakul *et al.*^31^ The negative predictive value of 98.1% from this study was similar to several studies with values of 97.7% - 99.8%.^27^,^28^ Gengsakul *et al* performed echocardiograms on all newborns in their study and could determine those without CHD confidently, unlike in the present study, which performed echocardiograms only on the Positive Screens and their comparative group. Albuquerque *et al* also liaised with a paediatric cardiology network that continuously searched for CHD even after discharge, a facility not available in this study. These methodological differences may account for the present study's observed lower specificity and negative predictive values.

The study employed a cheap and effective way to screen newborns for CHD. It does not rely on the top trained cadres of medical staff such as paediatric consultants or paediatric cardiologists. However, medical officers, house officers, and nurse practitioners can all be trained to perform this examination.^11^ This is particularly useful in poor-resourced centres with insufficient high cadre medical staff. However, the study was limited because not all newborns had an echocardiogram to ascertain the true negative of those with no evidence of cardiac pathology on CPE.

## Conclusion

Cardiovascular physical examination at birth is an effective and inexpensive screening tool for detecting CHD in newborns, which can easily be utilised in low-resource settings. Further studies based on a design where babies are tracked after discharge to ascertain their outcomes may be useful.
